# Sudden Unexpected Death in Epilepsy: Central Respiratory Chemoreception

**DOI:** 10.3390/ijms26041598

**Published:** 2025-02-13

**Authors:** Ayse S. Dereli, Auriane Apaire, Riem El Tahry

**Affiliations:** 1 Clinical Neuroscience, Institute of Neuroscience (IoNS), Université Catholique de Louvain, 1200 Brussels, Belgium; auriane.apaire@uclouvain.be (A.A.); riem.eltahry@saintluc.uclouvain.be (R.E.T.); 2 Walloon Excellence in Life Sciences and Biotechnology (WELBIO), WEL Research Institute, 1300 Wavre, Belgium; 3 Center for Refractory Epilepsy, Department of Neurology, Cliniques Universitaires Saint-Luc, 1200 Brussels, Belgium

**Keywords:** SUDEP, central respiratory chemoreception, epilepsy, retrotrapezoid nucleus, hypercapnia, neuropeptide, hypercapnic ventilatory response, galanin, PACAP, orexin, somatostatin

## Abstract

Sudden unexpected death in epilepsy (SUDEP) is a critical concern for individuals suffering from epilepsy, with respiratory dysfunction playing a significant role in its pathology. Fatal seizures are often characterized by central apnea and hypercapnia (elevated CO_2_ levels), indicating a failure in ventilatory control. Research has shown that both human epilepsy patients and animal models exhibit a reduced hypercapnic ventilatory response in the interictal (non-seizure) period, suggesting an impaired ability to regulate breathing in response to high CO_2_ levels. This review examines the role of central chemoreceptors—specifically the retrotrapezoid nucleus, raphe nuclei, nucleus tractus solitarius, locus coeruleus, and hypothalamus in this pathology. These structures are critical for sensing CO_2_ and maintaining respiratory homeostasis. Emerging evidence also implicates neuropeptidergic pathways within these chemoreceptive regions in SUDEP. Neuropeptides like galanin, pituitary adenylate cyclase-activating peptide (PACAP), orexin, somatostatin, and bombesin-like peptides may modulate chemosensitivity and respiratory function, potentially exacerbating respiratory failure during seizures. Understanding the mechanisms linking central chemoreception, respiratory control, and neuropeptidergic signaling is essential to developing targeted interventions to reduce the risk of SUDEP in epilepsy patients.

## 1. Introduction

Epilepsy is a neurological condition that affects an estimated 50 million people globally [1]. The annual cumulative incidence of epilepsy is 70 per 100,000 persons [2] and is associated with a heightened risk of premature death [3,4]. Mortality rates among people with epilepsy are up to 20 times higher than those in the general population [5,6,7,8], with an average reduction in life expectancy of about 11.84 years in males and 10.91 years in females [9]. This increased mortality is attributed to various factors, including psychiatric comorbidities, cardiovascular disorders, accidents, suicide, and sudden death [3].

Among the causes of death in epilepsy, sudden unexpected death in epilepsy (SUDEP) is particularly notable, ranking as the leading direct epilepsy-related cause of death and second only to stroke in terms of years of potential life lost in the United States [10]. SUDEP, by definition, refers to the unexpected death of a patient with epilepsy, with or without evidence for a seizure, not due to trauma, drowning, or status epilepticus (SE), with no other clear cause found during an autopsy [11,12]. The annual mortality rate due to SUDEP ranges from 0.4 to 2 per 1000 individuals with epilepsy [13,14,15]. Despite the severity of SUDEP, the underlying cellular and molecular mechanisms remain incompletely understood, and preventive therapies have not been established for those patients who fall into the high-risk group. Current research implicates central apnea, coupled with a cascade of autonomic dysregulation, as a key component of SUDEP’s pathophysiology [16]. Central apnea refers to the cessation of breathing due to centrally mediated mechanisms and is characterized with hypercapnia. Central CO_2_ chemoreceptors (CCR) detect blood CO_2_ levels to regulate breathing through their projections to lower-order regulatory regions, making them highly relevant to this pathology. However, despite their critical role, the involvement of central chemoreception in epilepsy remains to be fully established.

This review aims to explore the role of central respiratory chemoreception in the pathophysiology of SUDEP, providing a detailed molecular understanding of how disruptions in this process may contribute to sudden death in epilepsy. By shedding light on this under-researched area, the review seeks to inform future research and therapeutic strategies aimed at reducing the incidence of SUDEP in individuals with epilepsy.

## 2. Risk Factors for SUDEP

Some epileptic patients are more predisposed to SUDEP than others. Considerable efforts have been made to identify risk factors and develop stratification methods to predict SUDEP and apply preventative measures to mitigate the risk of death. These include the SUDEP-7 inventory [17], the SUDEP-3 inventory [18], individualized prediction tools [19,20], and the SUDEP safety checklist [21]. Some of these tools contain up to 22 risk factors [19]. The most recent SUDEP-CARE score [22] concentrated on seven critical risk factors strongly associated with SUDEP. These include generalized seizure frequency, nocturnal seizures, respiratory symptoms during or after a seizure, intellectual disability, current or past depression, the ability to alert someone of an oncoming seizure, and seizure-related falls.

Indeed, clinical research highlights that a higher frequency of tonic–clonic seizures per year is strongly correlated with increased SUDEP risk [23,24,25]. Additionally, the annual mortality rate due to SUDEP escalates to 4–9 per 1000 among epileptic patients that are refractory (drug resistant) [14,15,26]. In severe chronic refractory epilepsy patients who attend epilepsy referral centers, SUDEP is the leading cause of death, accounting for up to 50% of all fatalities [15]. Patients with multiple risk factors, such as those with refractory epilepsy experiencing generalized tonic–clonic seizures, face a significantly heightened risk [27]. Furthermore, nighttime seizures [24,25,28], particularly nocturnal generalized tonic–clonic seizures within the year preceding SUDEP, are associated with a 15-fold increase in risk [25]. Also, clinical studies in epilepsy monitoring units highlighted that an early postictal alteration in respiratory function induced by a generalized tonic–clonic seizure constitutes a critical risk factor for SUDEP [13]. These findings emphasize the necessity of close supervision and proactive management for patients with significant risk factors to mitigate the likelihood of SUDEP.

In addition to these, a long history of seizures and the duration of epilepsy also serve as notable risk factors [20]. Although SUDEP can affect all age groups, its incidence is high among young adults aged 20–45 years [29,30]. Also, in approximately 30% to 50% of SUDEP cases, genetic factors have been identified through postmortem testing [31,32]. In some cases, the underlying cause of epilepsy can be genetic; in others, patients with non-genetic forms of epilepsy may still possess genetic mutations [33] that may contribute to SUDEP.

## 3. Pathophysiology of SUDEP: Respiratory Arrest

Historically, SUDEP was predominantly attributed to cardiac arrest. However, recent research has uncovered a more complex and heterogeneous pathophysiology. In clinical studies investigating the incidence and mechanisms of cardiorespiratory arrests in epilepsy monitoring units (MORTEMUS), it was described that SUDEP generally follows a specific postictal sequence [16]. It typically begins with a centrally mediated disruption of respiratory and cardiac function, often triggered by a generalized tonic–clonic seizure. This sequence can result in immediate death or a brief period of partially restored cardiorespiratory function, which is subsequently followed by terminal apnea and cardiac arrest [13].

**Figure 1:**
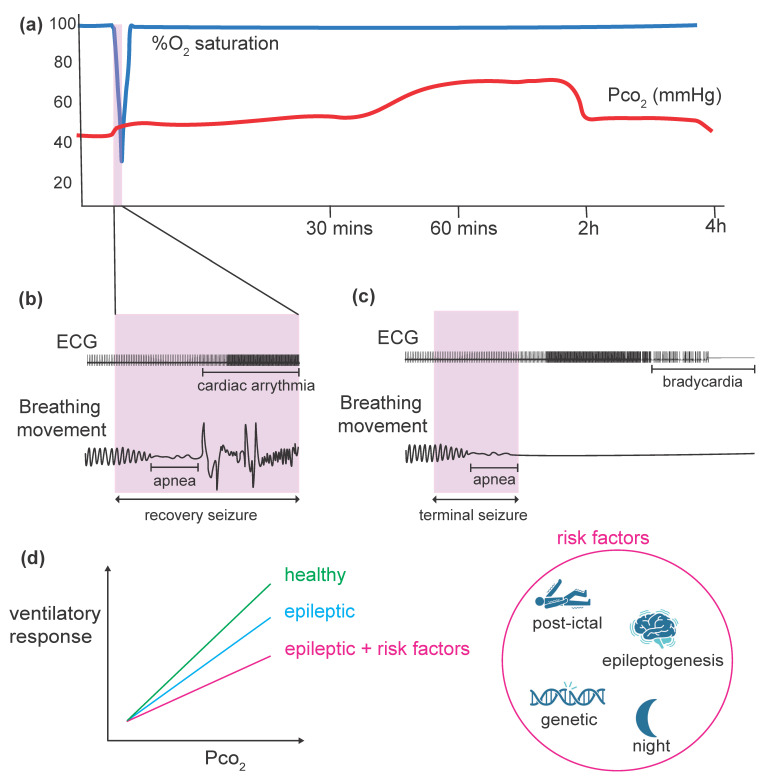
Fatal seizures are characterized by central apnea, hypoxia, and hypercapnia, and epilepsy is associated with an ablated hypercapnic ventilatory response (HCVR). (**a**) Percentage oxygen saturation (%O_2_, blue trace) decreases, and partial pressure of CO_2_ (Pco_2_, red trace) increases during a seizure (pink box). Elevated Pco_2_ persists for up to 4 h after the seizure, adapted from [37,45]. (**b**) A recovery seizure with apnea is followed by an increase in heart rate, adapted from [38]. (**c**) A terminal seizure, in which respiratory arrest is followed by secondary bradycardia, is shown, adapted from [45]. (**d**) HCVR is reduced in epileptic conditions, with more severe ablation observed in the presence of risk factors such as postictal timing, late-stage epileptogenesis, genetic factors, and nocturnal seizures. Summary trace gathered from [58,59,60,61].

Respiratory complications are reported in most witnessed and recorded SUDEP cases, including respiratory arrest, labored breathing, suffocation in a prone position, and laryngeal spasm [26,34,35]. The historical understanding of respiratory issues associated with SUDEP dates back to 1899, when Hughlings Jackson observed that patients would stop breathing and turn blue following generalized seizures, indicating blood deoxygenation [36]. Even during focal unaware seizures, a reduction in oxygen saturation to as low as 30% has been documented in epileptic patients [37] highlighting the significance of respiratory disturbances during SUDEP (Figure 1a).

There is increasing evidence that respiratory problems often precede cardiac arrhythmias in SUDEP. Some seizures are characterized by apnea-induced hypoxemia without associated cardiac arrhythmia [38,39] (Figure 1b). Additionally, video-EEG recordings rarely show cardiac problems as the primary cause of SUDEP, instead pointing to respiratory arrest [34,35,40,41,42,43]. Moreover, prolonged generalized EEG suppression in SUDEP [44] indicates that it is more expected for generalized seizures to affect breathing directly and not necessarily the cardioregulation, as the heart can function autonomously. Recent animal studies supported these findings by directly showing that central apnea often precedes terminal bradycardia following terminal seizures (Figure 1c) in genetic-induced [45,46,47,48,49,50,51], electroshock-induced [50], and chemically induced [52] models of SUDEP. Hence, respiratory arrest is suggested to be the primary cause of SUDEP.

When it comes to cardiac arrest, for a long time, the decrease in heart rate postictally was believed to result from increased vagal output [53]. However, further investigation using muscarinic receptor antagonists has provided evidence that bradycardia is indeed caused by apnea-induced hypoxia rather than parasympathetic dominance [45]. Consequently, the primary cause of postictal death is increasingly being attributed to central apnea or respiratory arrest, as reviewed multiple times [54,55], emphasizing the critical role of postictal respiratory dysfunction in many cases of SUDEP. Nevertheless, it is important to note that SUDEP likely involves multifactorial mechanisms, including impaired arousal mechanisms from sleep, as suggested by recent findings [56,57].

## 4. Animal Models of SUDEP to Study Respiratory Dysfunction in Epilepsy

### 4.1. Choice of Animal Models

Animal models are important tools to understand the underlying mechanisms for central apnea and respiratory arrest in SUDEP. A wide variety of animal models have been developed to date [62], each tailored to address specific research questions. This diversity underscores the absence of a universal gold standard model for SUDEP. When investigating respiratory dysfunction, it is essential to consider previously identified risk factors alongside the established definition of SUDEP, including the following:

- Unexpected death with or without evidence for a seizure, not due to other causes (including SE);
- Whether the seizures are acute or chronic;
- The duration and history of epilepsy;
- The frequency of seizures;
- The type of seizures;
- Whether the seizures are spontaneous;
- Genetic predisposition;
- Nocturnal seizures;
- The absence of other neurological, neurodevelopmental, or non-neurologic pathology.

Most animal model studies on SUDEP to date have been acute, primarily focusing on the periods surrounding terminal seizures, specifically the ictal and postictal phases [45,49,52,63,64,65,66,67]. However, SUDEP occurs in individuals with chronic epilepsy and in rare cases happens without seizure [11,12], and it is still unclear why long-term and frequent seizures are more prone to fatal outcomes.

Frequent seizures could potentially have an accumulative effect on normal respiratory function (interictal breathing), progressively increasing the risk of respiratory failure and thus the likelihood of SUDEP. In favor for this idea, numerous direct and indirect neural pathways link the cortex to brainstem respiratory centers [68,69], and evidence suggests that seizure activity can spread to lower brain regions, leading to depolarization in areas like the brainstem [64,65,70,71,72,73]. Hence, the normal functioning of lower order brain regions can be affected and impaired over time under the chronic effect of seizures. Indeed, functional connectivity studies using magnetic resonance imaging (MRI) have identified disruptions in brainstem arousal centers in patients with temporal lobe epilepsy (TLE) [74]. Additionally, cumulative neuronal changes were observed in brainstem centers that control breathing in postmortem SUDEP patients [75,76,77,78,79,80]. Some epilepsy patients also experience breathing difficulties not only during seizures but also interictally, further indicating that chronic epilepsy can lead to long-term alterations in respiratory regulation, resulting in persistent respiratory symptoms [81]. Hence, while acute epilepsy models provide valuable insight into the immediate cardiorespiratory complications of terminal seizures, they are limited in their ability to mimic the chronic effects of epilepsy. As a result, chronic spontaneous epilepsy models are essential for understanding how long-term seizure activity affects autonomic regulation and contributes to the development of SUDEP.

In chronic epilepsy models, the history, frequency and type of seizures should also be taken into account when deducing conclusions about SUDEP pathology. Furthermore, animal models can be classified based on the induction of epileptic seizures including chemical, audio, electrical stimuli, or genetic predisposition (Table 1). While most of these models can be used in both acute and chronic setups, not all are characterized with spontaneous seizures. SUDEP rates also vary between models and in some cases depend on the intensity of the stimulus, like the dose and administration route [82]. Among all the animal models, here, we will provide a brief introduction on the ones used to explore respiratory symptoms in SUDEP.

### 4.2. Animal Models Used to Study Pathophysiology of SUDEP

Kainic acid and pilocarpine are chemically induced, chronic models of TLE characterized with tonic–clonic seizures [83,84,85,86]. They can be administered systemically or intrahippocampally and follow similar patterns of events that happen in TLE. First, they manifest a stage of SE for 6–12 h shortly after administration. After SE follows a latent period of 2–3 weeks, which is a seizure-free time interval, where there is reorganization of neuronal networks. Spontaneous seizures begin to appear gradually increasing in the number of seizures per day over time. There is usually a high death toll during SE; however, by definition, these cannot be defined as SUDEP. Rats that survive SE can be used as chronic models, which are also associated with a lower survival rate with no obvious causes of death [87,88], as is the case in SUDEP. Nevertheless, the presence of an increasing frequency of tonic–clonic seizures makes them appealing to study SUDEP. Moreover, these chemically induced models manifest autonomic dysfunctions, including respiratory dysregulation, as is the case in SUDEP. For example, in KA injected rats, seizures are associated with massive increases in parasympathetic (vagus nerves) and sympathetic (cervical sympathetic ganglion > renal nerve > splanchnic nerve) activity [89]. KA rats have also shown suppression of phrenic nerve activity [90], which is the only motor nerve innervating the diaphragm and is regulated by the central respiratory centers. Indeed, anesthetized setups with induced seizures through an injection of kainic acid were characterized with central apneas, indicating a compromise in central breathing regulation [65,67,91]. Also, epileptogenesis (the process by which the brain network is altered toward increased seizure susceptibility, thus having an enhanced probability to generate spontaneous recurrent seizures) in KA rats exhibits increased severity with duration and reaches a plateau in the number of seizures/day after about 4 months [92]. The epileptogenesis studies in a pilocarpine model reveal that the number of seizures reach maximum after 1.5–3 months depending on the dose; then, they decrease afterwards [93]. Since SUDEP increases with the severity of epilepsy, these models allow for the study of the progressive autonomic instability to draw conclusions about interictal pathophysiologies [60].

Audiogenic models include Wistar audiogenic rats (WARs) and dilute brown agouti coat color (DBA) mice. In both cases, an audiogenic stimulation triggers tonic–clonic seizures [94,95]. Hyperexcitation starts at the level of the inferior colliculus of the midbrain and propagates to cortical and other subcortical structures, including the brainstem, the periaqueductal gray complex, and the amygdala [96,97,98]. DBA mice develop 100% postictal respiratory arrest and death after a few days of stimulation [48,49]. This makes them a controlled model for SUDEP, in which a reproducible trend of autonomic alterations happens, replicative of seizure-induced death in humans [94]. Hence, DBA mice are attractive to investigate ictal and postictal events during SUDEP. Unlike DBA, WARs are not characterized with seizure-induced death, but they still develop respiratory impairment, with changes in baseline breathing [95,99]. Both these models can also be used as chronic seizure models by repetitive audiogenic stimulation. Chronic WARs, also called kindled WARs, are obtained with two audiogenic stimulations/day for about 2 weeks and develop also limbic seizures [95,100]. There are two types of DBA mice (DBA/1 and DBA/2). While DBA/2 mice are only susceptible to seizures at a relatively young age, postnatal day (P)21 to P28, DBA/1 mice are susceptible until P100 [49]. Since both DBA mice develop respiratory arrest following audiogenic stimulus, they require resuscitation to survive from seizures and become chronic [49]. The high rate of seizure-mediated deaths makes these models attractive to study SUDEP.

The amygdala rapid kindling (ARK) model is another model for TLE. Kindling involves multiple electrical stimuli to the amygdala. This initiates focal seizures with minimal behavioral response that changes progressively over repetitions and begins spreading to other brain regions and becomes generalized afterwards [101,102]. For spontaneous seizures to occur in ARK, a mean of 348 stimulations are required [103]. Spontaneous seizures persist for as long as 7 months following termination of the stimulation. ARK models are also characterized with cardiorespiratory impairments, with changes in baseline breathing [101] and heart rate [104,105]. While the occurrence of SUDEP is not documented in the ARK model, it is a model of chronic epilepsy with spontaneous generalized tonic–clonic seizures to investigate the epileptogenesis.

There are also genes that can increase susceptibility to epilepsy and respiratory dysfunction precipitating to SUDEP. Postmortem tests identified genetic factors in 30% to 50% of SUDEP cases [31,32]. These include the *Scn1a* gene (that codes for sodium channels) and *Kcna1 and Kcnq2* genes (coding potassium channels).

Dravet syndrome (DS) is associated with a loss-of-function (LoF) missense mutation at the *Scn1a* gene and is a severe form of epilepsy with a high incidence of SUDEP [106,107,108]. The *Scn1a* gene encodes for NaV1.1 voltage-gated sodium channel. The mutation of NaV1.1 causes failure of excitability of hippocampal GABAergic inhibitory interneurons leading to hyperexcited epilepsy syndrome [109]. The mouse model of DS recapitulates many aspects of the clinical condition and offers a research tool for understanding mechanisms of SUDEP in DS. Global heterozygous knockout (KO) of *Scn1a* is a popular model for DS and leads to an early onset of seizures, starting at P21 [109,110], with high susceptibility to hyperthermia [111]. The seizures are spontaneous with age-dependent severity [111]. The deaths are spontaneous, sporadic, and premature; by P28, 50% of mice die following a tonic–clonic seizure [110]. Furthermore, these mice have autonomic symptoms, including reduced interictal heart rate variability and bradyarrhythmia [110]. SUDEP, in these mice, is prevented by administration of atropine, indicating that there is parasympathetic hyperactivity during seizures, leading to death [110]. Moreover mouse models of DS carrying a missense mutation in the *Scn1a* gene, conditionally in inhibitory neurons, exhibit hypoventilation and frequent apneas under baseline conditions [59]. Therefore, these models serve as a valuable representation of a genetic type of SUDEP with aberrant autonomic symptoms.

Mutations at the level of voltage-gated potassium channel subfamilies are also associated with epilepsy, respiratory dysfunction, and SUDEP in humans [112]. There are various mice models that replicate these mutations such as *Kcna1* gene KO mice, which lack Kv1.1 voltage-gated potassium channel that controls action potential firing properties in brain and heart [113]. These mice experience spontaneous seizures starting around P14 [114,115]. Median survival rate for *Kcna1* KO mice is 47 days [116] with deaths occurring between P14–70 in about 75% of animals [117]. These mice display apnea during seizures and present baseline breathing irregularities, including mild tachypnea, increased respiratory variability, an absence of post-sigh apneas, and frequent hypoxia [51,118]. The respiratory dysfunction progresses with age in *Kcna1* gene KO mice [118].

Similarly, mutations in the *Kcnq* gene, which encodes for the Kv7 family of potassium channels, are associated with specific types of epilepsy linked to human SUDEP [119]. Kv7 channels generate a slow-activating potassium current, also known as the M-current, that plays a crucial role in regulating neuronal excitability by preventing excessive neuronal firing. Both LoF [120] and gain-of-function (GoF) [121] mutations of *Kcnq2* are seen to result in neuronal hyperexcitability, increasing susceptibility to spontaneous firing and seizures. In mice, *Kcnq2* GoF mutations carrying the R201C variant in excitatory forebrain neurons are characterized with tonic–clonic seizures with premature lethality, beginning as early as P40, with almost complete lethality by P100 [121]. Humans with GoF suffer from neonatal-onset encephalopathy, myoclonus, multifocal seizures later in life, respiratory dysfunction (perinatal respiratory failure and/or chronic hypoventilation), developmental problems, and early mortality [122]. When this mutation is induced in *Phox2b*-expressing respiratory neurons in mice, profound hypoventilation is observed but not necessarily death [61]. In summary, mice with Kv1 and newly emerging Kv7 mutation are models for SUDEP with spontaneous seizures and respiratory anomalies while remaining genetic models for SUDEP.

### 4.3. How Close Are These Models to Human SUDEP?

Although these models have significantly advanced our understanding of the mechanisms underlying respiratory dysfunction during seizures and SUDEP, they also present limitations in accurately reflecting human clinical observations. As mentioned earlier, one major limitation is that most SUDEP studies in animal models rely on acute seizures [45,49,52,63,64,65,66,67], which may not fully replicate the chronic nature of human epilepsy or SUDEP. This challenge makes it difficult to study the long-term effects of seizures and to develop chronic therapies for SUDEP.

Among the chronic models reviewed here, WAR, DBA, and ARK require repeated induction to provoke seizures [48,49,95], a characteristic that differs from human cases where seizures typically occur spontaneously. Chronic KA, pilocarpine and genetic epilepsies can be preferred for spontaneously occurring seizures. However monitoring large numbers of animals over extended periods to capture SUDEP incidence remains challenging, particularly in chronic KA [92] and pilocarpine rats [84], where the SUDEP incidence is relatively low compared to genetic epilepsy models [109,110,111,115,116,121,123]. Furthermore, while these models exhibit spontaneous seizures, their initial seizure induction is chemically triggered, which does not accurately reflect the natural occurrence of seizures in humans.

Additionally, KA and pilocarpine models, which serve as models for TLE, typically begin with a phase of SE [84,92]. This differs from human clinical circumstances in two ways. First, SE is more common in children [124,125], whereas in animal studies, adult rodents are often used, as younger animals have higher mortality rates and longer latent periods [84]. Second, in human clinical settings, SE onset is not necessarily associated with TLE [126,127]. Moreover, although TLE is strongly linked to refractory epilepsy [128,129], there is no conclusive evidence that these chemically induced models exhibit drug resistance.

Genetic models may provide a closer approximation of genetic epilepsies; however, it is important to acknowledge that not all clinical SUDEP cases have a genetic basis [31,32]. Although their high SUDEP incidence makes them convenient for experimental studies, SUDEP remains a rare occurrence in human clinical settings [10].

Furthermore, the anatomical origin of seizures in some models, such as audiogenic models (DBA and WAR), originates in the brainstem, which differs from the primary seizure focus in human epilepsy [95,96,97,98,128]. Additionally, in DBA, genetic, and acute KA or pilocarpine models, death occurs immediately following severe seizures. However, in humans, SUDEP may or may not occur after seizures [11,12], and seizures themselves are not always fatal.

Therefore, careful consideration of each model’s characteristics, strengths, and limitations is essential when interpreting preclinical data and translating findings into diagnostic and therapeutic applications for patients [62].

**Table 1:** Animal models of SUDEP.

Stimulus	Model	Species	Acute/Chronic	Spontaneous/ Induced	Seizure Type	Death Rate	References
Chemical	Kainic acid	Rat/Mouse	Acute	Spontaneous	SE with generalized seizures, including tonic–clonic	High *	[65]
			Chronic	Spontaneous	TLE with generalized seizures, including tonic–clonic	Low *	[92]
	Pilocarpine	Rat/Mouse	Acute	Spontaneous	SE with generalized seizures, including tonic–clonic	High *	[84]
			Chronic	Spontaneous	TLE with generalized seizures, including tonic–clonic	Low *	[84]
Audio	Wistar audiogenic rat (WAR)	Rat	Acute	Induced	Tonic–clonic seizures (acute)	Low	[95]
			Chronic (audiogenic kindling)	Induced	TLE	Low	[95]
	DBA/1	Mouse	Acute or Chronic (with resuscitation)	Induced	General convulsive seizures	High (90–100%) with postictal respiratory arrest after 3–4 days of stimulus.	[49]
Electrical	Amygdala rapid kindling (ARK)	Rat/Mouse	Chronic after >300 stimuli	Induced (Spontaneous seizures may occur)	TLE with generalized seizures, including tonic–clonic	Low	[103,129]
Genetic	*Scn1a* (LoF)	Mouse	Chronic (until premature death)	Spontaneous/ Heat-induced	Dravet syndrome with tonic–clonic seizures	High premature deaths (46%, by PD28)	[109,110,111]
	*Kcna1* KO	Mouse	Chronic (until premature death)	Spontaneous	Early-onset generalized spontaneous tonic–clonic seizures (up to ~24/d beginning at P14)	High premature deaths (75%, P14–70)	[115,116,123]
	*Kcnq^R201C^* (GoF)	Mouse	Chronic (until premature death)	Spontaneous	Tonic–clonic seizures Absence seizures	High premature deaths starts at ~P30 and reaches 100% by ~P80	[121]

* depends on the dose, administration route, and strain of the anima.

## 5. Disruption of Central Chemorespiratory Mechanisms in SUDEP

**Figure 2:**
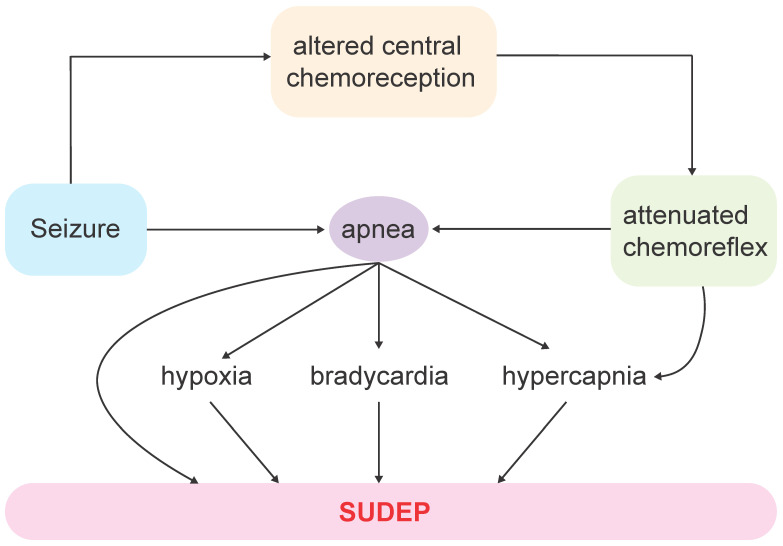
A schematic summary of the cardiorespiratory symptoms and the contribution of central chemoreception in the SUDEP cascade. Repetitive seizures induce alterations in the central chemoreceptive centers, leading to impaired chemoreflex. This dysfunction contributes to central apnea, exacerbating hypoxia and hypercapnia. Hypoxia is proposed to cause cardiac asystole, culminating in SUDEP.

Research indicates that central apnea during seizures is associated with increased levels of end-tidal and transcutaneous CO_2_ in human patients [45,130] and animals [131] (Figure 1a). While the rise in end-tidal CO_2_ persists for 7 min [130], transcutaneous CO_2_ levels remain elevated for up to 4 h post-seizure [45] in humans. These findings suggest a prolonged suppression of CO_2_ chemoreception (Figure 2), which is the brain’s mechanism for detecting changes in the partial pressure of CO_2_ in the blood (Pco_2_) and regulating breathing accordingly. Patients with epilepsy who experience sustained postictal CO_2_ elevation are at a higher risk of SUDEP [45], highlighting the need for a detailed investigation of CO_2_ chemoreception.

A common method to measure CO_2_ chemoreception is the ventilatory response to elevated blood CO_2_, known as the hypercapnic ventilatory response (HCVR). A series of studies indicate that HCVR decreases in epileptic conditions (summarized in Table 2) with the influence of several risk factors, including the timing in relation to the seizure, epileptogenesis, genetic factors, and the vigilance state (Figure 1d).

In epilepsy monitoring units, rebreathing techniques used to measure interictal HCVR have revealed significant interindividual differences. Some patients exhibited severe hypoventilation, leading to prolonged end-tidal hypercapnia, indicating reduced HCVR and, consequently, diminished respiratory CO_2_ chemosensitivity. Furthermore, the interictal HCVR was correlated with postictal end-tidal CO_2_ concentration and duration following generalized convulsive seizures, revealing an inverse relationship between these parameters [81]. This suggests that certain patients are more prone to prolonged postictal CO_2_ elevation after generalized convulsive seizures. Further studies extended these findings by testing HCVR after seizures, revealing a blunted CO_2_ sensitivity postictally for up to three hours following both focal and generalized seizures, thereby potentially increasing the risk of severe respiratory depression and SUDEP [58] (Figure 1d and Figure 2).

Similarly, chronic animal models of epilepsy demonstrate impaired ability to adjust to variations in Pco_2_. In a study on epileptic rats induced by pilocarpine and subjected to artificial hyperventilation using a respiratory pump to elevate blood CO_2_, it was found that anesthetized epileptic rats had an altered capacity to compensate for changes in arterial CO_2_. However, when peripheral chemoreceptor responses were assessed in the same animals using potassium cyanide injections, no significant differences were observed between epileptic and healthy groups. This suggests that the observed alterations in CO_2_ sensitivity were not of peripheral origin [132]. Seventeen years later, these conclusions were supported by another study measuring ventilatory response to hypercapnic and hypoxic exposures in pilocarpine rats. The results have indicated a decrease in central (hypercapnia) but not peripheral (hypoxia) chemosensitivity 15 and 30 days post-status epilepticus (post-SE) in awake conditions [133]. Testing HCVR in kainic acid-induced mice also showed decreased HCVR for up to five weeks post-SE, with partial recovery by the seventh week [60] (Figure 1d). Amongst audiogenic models, naïve WARs exposed to hypercapnia revealed decreased chemosensitivity compared to healthy controls [134]. However, when WARs were chronically induced by audiogenic kindling (10 days), their HCVR remained diminished but was not significantly different from naïve WARs [99]. This suggests a genetic predisposition in WARs to reduced chemosensitivity, which seizures do not exacerbate. Moreover, the ARK model of TLE also demonstrated reduced HCVR in kindled rats (10 stimulation/day for 2 days) compared to healthy ones [101].

Similar observations were seen in genetic models of SUDEP. In the model of DS with a mutation on *Scn1a* gene globally [58] or conditionally in interneurons [59], a decrease in HCVR was observed at P15-P34 (Figure 1d). These mice usually die prematurely (starting at P15) [59], explaining why the experiments were conducted at a young age. Further, supporting these observations, genetically derived epileptic mice with a GoF mutation in *Kcnq2* gene in the retrotrapezoid nucleus (RTN) displayed decreased central chemoreflex during the light/inactive phase [61] (Figure 1d). The RTN being a CO_2_-sensitive population, this aligns with the idea that SUDEP risk may be heightened at night, drawing attention to the role of central chemoreceptive mechanisms in SUDEP. These findings suggest that dysregulation at the level of central chemoreception may contribute to epilepsy-related respiratory arrest, resulting in mortality.

Conversely, in a few studies, an increase in CO_2_ sensitivity was observed in epileptic conditions (Table 3). For instance, in pilocarpine-induced rats with chronic epilepsy, a decrease in latency to awaken from obstructive sleep apneas during REM sleep was observed, indicating enhanced chemosensitivity and arousal mechanisms [135]. Supporting this, another study on pilocarpine-induced epileptic rats reported an enhanced ventilatory response to hypoxia and hypercapnia in some epileptic rats compared to the controls [136]. The pilocarpine model is suggested to become chronic after approximately 44 days [137,138]. These studies, conducted 6–12 weeks post-SE, propose that there might be potential neuroplasticity in brain regions involved in respiratory control, arousal mechanisms, and autonomic modulation enhancing chemosensitivity. However, the latter study also identified a low steady respiratory pattern and a sharp decrease in interictal oxygen consumption, which, contrary to the findings, might indicate decreased chemosensitivity. Since oxygen consumption reflects respiratory gas exchange during ventilation, its reduction could point to ventilatory pauses or gasping, contradicting the hypothesis of increased chemosensitivity. Despite this contradiction, a recent study using *Kcna* KO mice similarly demonstrated an increase in HCVR [139]. It is important to note that comparing findings across different studies is challenging due to variations in experimental models, strains, the timing of respiratory recordings, and the severity of hypoxia and hypercapnia exposures. Even within the same model, differences may arise from the strain or the route of administration. Nevertheless, collectively, these studies suggest that the ventilatory response to hypercapnia is altered in SUDEP models, highlighting the need for further research to understand the underlying mechanisms.

**Table 2:** Suggested involvement of decreased central respiratory CO_2_ chemosensitivity in SUDEP (n/a: not available).

Species	Model	Condition	Epileptogenesis	Evidence	Reference
Human	n/a	n/a	n/a	Increased postictal transcutaneous CO_2_ in human epileptic patients	[45]
	n/a	n/a	n/a	Increased postictal end-tidal CO_2_ in human epileptic patients	[130]
	n/a	n/a	n/a	Decreased interictal HCVR in epilepsy monitoring unit patients	[81]
	n/a	n/a	n/a	Blunted HCVR after focal and GCSs vs interictal in human epileptic patients	[58]
Rats	WAR: naïve	Chronic unanesthetized	P30–40	Decreased HCVR	[134]
	WAR: naïve and audiogenic kindled for 10 days (2 stimuli/day)	Chronic unanesthetized	Straight after kindling	Decreased interictal HCVR in both naïve and kindled WARs with no difference in between	[99]
	ARK Wistar: acute electrical stimulation of basolateral amygdala for 2 consecutive days (10 stimuli/day)	Chronic unanesthetized	10 days after ARK	Decreased HCVR	[101]
	Pilocarpine Wistar (intraperitoneal)	Chronic anesthetized	6–10 months post-SE	Alteration in ability to compensate for changes in arterial CO_2_	[132]
	Pilocarpine Wistar (intrahippocampal)	Chronic unanesthetized	15–30 days post-SE	Decreased interictal HCVR	[133]
Mouse	Kainic acid (intrahippocampal)	Chronic unanesthetized	5–7 weeks post SE	Decreased interictal HCVR 5 weeks post-SE induction with its partial recovery at week 7	[60]
	*Scn1a* mutation	Chronic unanesthetized	P22–P34	Blunted postictal HCVR (normal interictal)	[58]
	*Scn1a* mutation	Chronic unanesthetized	P15	Decreased interictal HCVR	[59]
	*Kcnq2* GoF	Chronic unanesthetized	P30–50	Decreased interictal HCVR during the light/inactive phase	[61]

## 6. Mechanisms for Disrupted Chemoreception

Although there is evidence supporting decreased central chemoreception in chronic epilepsy, it remains uncertain whether elements of respiratory control are compromised in epileptic conditions. Some hypotheses suggest that repeated seizure activity disrupts respiratory control through feed-forward mechanisms, such as spreading depolarization [64,71,72] or by activating inhibitory subcortical projections to brainstem respiratory centers [70,73]. During terminal seizures in KA rats, the timing of brainstem depolarization suggests that it is a consequence, rather than a cause, of respiratory collapse [65]. Therefore, additional mechanisms—such as the impairment of central chemoreceptive networks—could contribute to respiratory dysfunction.

**Figure 3:**
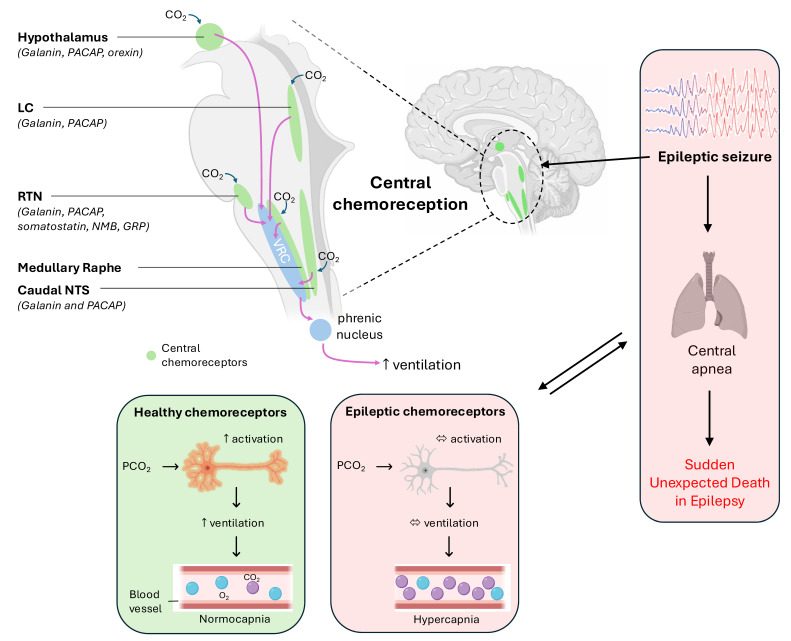
Schema depicting the role of central chemoreception and its dysfunction in the context of epilepsy, highlighting the involvement of neuropeptides. Central chemoreceptors, including the hypothalamus, and key brainstem regions, including the RTN, LC, medullary raphe, and caudal NTS, are activated in response to CO_2_ (blue arrows). Signals from central chemoreceptors project to the VRC, which sends information to the phrenic nucleus, which drives increased ventilation in response (neuronal projections represented by purple arrows). Central chemoreceptors contain neuropeptides: galanin, PACAP, orexin, somatostatin, NMB, and GRP, which are involved in the mechanism of central chemoreception. In healthy individuals (green box), central chemoreceptors respond to increased levels of PCO_2_ by enhancing respiratory drive, maintaining normocapnia. However, in epilepsy (red boxes), the impaired activation of these chemoreceptors can lead to reduced ventilatory responses, resulting in hypercapnia and an increased risk of central apnea, which is strongly associated with SUDEP (associations are indicated by black arrows). Abbreviations: LC: locus coeruleus, NTS: nucleus tractus solitarius, PACAP: pituitary adenylate cyclase-activating polypeptide, NMB: neuromedin B (NMB) and GRP: gastrin releasing peptide, PCO_2_: partial pressure of carbon dioxide, RTN: retrotrapezoid nucleus, SUDEP: sudden unexpected death in epilepsy, VRC: ventral respiratory column. Created with BioRender.

Central chemoreception involves detecting changes in Pco_2_ in the brain and regulating breathing accordingly. This process is modulated by neurons from different nuclei in the lower brainstem called central CO_2_ chemoreceptors (CCRs), ensuring Pco_2_ levels remain within a narrow range [140,141]. The RTN, raphe nucleus, the nucleus of the solitary tract (NTS), locus coeruleus (LC), and hypothalamus play crucial roles in this regulation [142] (Table 4). Once activated by elevated Pco_2_, their projections to respiratory regulatory regions, including the ventral respiratory column (VRC), allows them to modulate breathing to maintain blood CO_2_ homeostatis [143] (Figure 3).

### 6.1. Retrotrapezoid Nucleus

The RTN is of particular interest, as it regulates breathing directly [144,145] in response to changes in CO_2_/H^+^ levels and functions as a key locus of respiratory control by integrating information from all the other chemoresponsive regions [142]. CCR properties of RTN neurons have been comprehensively characterized. Their CO_2_ sensitivity via intrinsic proton receptors and the underlying cellular and molecular mechanisms have been investigated very extensively. Located within the ventrolateral medulla (VLM), RTN neurons are primarily glutamatergic, paired-like homeobox 2B-positive (Phox2b+), and tyrosine hydroxylase-negative (TH−). The RTN contains proton-sensitive neurons [146], which are vital for maintaining central respiratory control. Impairments in RTN chemosensitivity are implicated in fatal conditions such as congenital central hypoventilation syndrome (CCHS) [147,148,149] and sudden infant death syndrome (SIDS) [150]. Both syndromes involve genetic mutations (*Phox2b* and *Pacap*, pituitary adenylate cyclase-activating peptide) linked to severe hypoventilation and apneas, which are exacerbated during sleep. Some CCHS patients even require mechanical ventilation during sleep. Since SUDEP is also associated with apneas and increased risk during sleep, Pansani et al. (2016) highlighted the importance of investigating changes in chemosensitive RTN neurons as a potential mechanism underlying SUDEP [151].

A number of studies have addressed the role of the RTN in SUDEP pathophysiology. For instance, naïve WARs, which exhibit a decreased ventilatory response to CO_2_, show reduced numbers of Phox2b-expressing RTN neurons and reduced activation of these neurons in response to hypercapnia (c-Fos immunohistochemistry) [99]. Although these rats were not induced to have seizures, the same group repeated similar experiments in ARK rats, a different model of epilepsy. These rats exhibited tonic–clonic seizures and also showed a decreased ventilatory response to CO_2_, with reduced CO_2_-induced c-Fos expression in the RTN [101]. Further studies using Ca^2+^ imaging in kainic acid-induced chronic epileptic mice revealed that the firing profile of RTN neurons in response to CO_2_ was abolished in freely moving mice [60]. Moreover, brainstem slices from DS mice showed altered RTN electrical activity in response to CO_2_/H+ [59]. Together, these studies suggest that a reduced neuronal population, diminished CO_2_-induced activity, and altered firing profile of the RTN contribute to impaired central chemoreception in epileptic models, potentially leading to premature death.

Although Phox2b mutations are not considered major risk factors for SUDEP [152], epilepsy-related gene mutations can affect the RTN. Immunohistochemistry in healthy mice shows that the RTN expresses the Kv1.1 protein [153], suggesting that it is a substrate for the epilepsy-related *Kcna1* mutation, potentially affecting its excitability. In *Kcna1* KO mice, significant astrocytosis and microgliosis were observed in chemosensitive regions, including the RTN, suggesting that seizure-related brain injury may contribute to observed respiratory abnormalities [153]. Similarly, patch-clamp studies on RTN slices revealed that the RTN expresses *Kcnq* transcripts, another epilepsy-related gene [154]. The administration of KCNQ antagonists increased basal activity and CO_2_ responsiveness of RTN neurons, while KCNQ agonists silenced them, indicating that KCNQ channels are key determinants of spontaneous RTN neuron activity in vitro [154]. This suggests that KCNQ channels may represent a common molecular basis for respiratory deficits in certain types of epilepsy. Furthermore, the effect of KCNQ channels on RTN spontaneous activity was absent in awake animals, indicating that this association is state dependent [61]. More research is needed to determine whether this contributes to increased SUDEP risk during sleep. Recent in situ hybridization studies have shown that the RTN expresses the *Kcnq2* isoform but not *Kcnq3* [61]. Impairments in the RTN may underlie the respiratory issues experienced by patients with *Kcnq2* gene mutations, such as those characterized by a GoF variant, R201C [122]. Functional studies on mice further support this idea, showing that *Kcnq2* deletion or expression of the GoF R201C variant in Phox2b-expressing neurons leads to increased baseline breathing or decreased central chemoreflex, respectively, in mice during their inactive (light) state [61]. These findings suggest RTN neurons could be key substrates for genetic epilepsy-related breathing abnormalities.

### 6.2. Raphe

Raphe neurons are identified by their expression and release of serotonin (5-HT). Caudal raphe nuclei are known to innervate neuronal groups closely implicated in the regulation of breathing [155,156]. Serotonergic neurons can have chemosensory properties and provide excitatory drive towards the respiratory rhythm generation through several 5-HT receptor subtypes [155,157,158].

Immunohistochemical studies on postmortem human SUDEP cases, showed that there is decreased 5-HT synthesizing enzyme (Tryptophan hydroxylase 2, TPH2), 5-HT presynaptic transporter (SERT) in medullary raphe, indicating that there is loss of serotonergic neuronal synthesizing capacity and reuptake mechanisms in SUDEP patients [80]. MRI studies show that there is medullary atrophy, prominently in the raphe and VLM in SUDEP cases [159,160,161]. These findings were in concert with animal models of epilepsy associated with respiratory dysfunction. For instance, WAR, which are characterized with reduced basal breathing rate, have a decreased number of medullary serotonergic neurons (raphe pallidus and obscurus) [99]. Hence, SUDEP or epilepsy with respiratory impairments are characterized with decreased serotonergic transmission.

Impairment of the 5-HT system is proposed to contribute to decreased HCVR in epileptic situations. In WAR and ARK with altered HCVR, there is a decrease of interictal CO_2_-activation in 5-HT neurons (c-Fos immunohistochemistry) [99,101]. Lmx1bf/f/p mice, which lack more than 99% of 5-HT neurons [162], not only have impaired HCVR [163] but also arousal response to hypercapnia during sleep [56,57]. Hence, 5-HT neurons are suggested to be important to respond to hypercapnia, which becomes important during fatal seizures at night.

Moreover, multiunit and single-cell recordings showed decreased population firing of the medullary and midbrain raphe neurons during the ictal and postictal periods in anesthetized rats induced with intrahippocampal electrical stimulation [164]. The markedly suppressed firing of 5-HT neurons was in concert with decreased respiratory rate, tidal volume, and minute ventilation during and after seizures and supports their possible role in simultaneously impaired cardiorespiratory function in seizures [164]. Conversely, MRI studies on DBA/1, straight after audiogenic seizure-induced respiratory arrest, indicate increased activity at raphe nuclei [96]. The activation of 5-HT neurons could be dependent on the type of seizures. In fatal seizures, activation of serotonergic mechanisms could be favored as a compensatory mechanism for apnea. However, they were not sufficient to prevent death, as there probably was not enough 5-HT neurons and innervations. These studies clearly indicate that impairment of the 5-HT system is associated with an increased risk of respiratory dysfunction in epileptic situations and may contribute to SUDEP.

Moreover, in epileptic conditions, the excitatory drive to respiratory regions by serotonergic neurons has also been shown to be affected. In postmortem SUDEP brainstems, there was a significant reduction of SERT and TPH2 in the VLM [80]. In a chronic pilocarpine-induced epileptic model, rats that experienced a sharper decrease in oxygen consumption were characterized by decreased 5-HT levels in both the NTS and VLM [136]. These findings suggest that 5-HT delivery to respiratory regions is impaired. Conversely, gene expression studies showed that in WAR and acute DBA/2 mice, which exhibit fatal respiratory symptoms, there was an increase in transcript levels for TPH2 and SERT in the brainstem [136]. This increase in mRNA may represent an adaptive mechanism to compensate for the reduced protein levels. However, it is important to note that the increased transcript levels were observed in whole brainstem samples using quantitative PCR, while the decreased protein levels were found in specific regions through immunohistochemistry. Further research is required to clarify which stage of 5-HT synthesis is impaired. Overall, these findings again suggest that alterations in the 5-HT system may play a role in epilepsy with severe respiratory phenotypes.

There are several fatal epilepsy models derived from mutations in the serotonergic system. For example, Lmx1bf/f/p mice exhibit a decreased seizure threshold in response to maximal electroshock (MES) and an increased rate of seizure-induced mortality due to respiratory failure [50]. In these mice, seizure-induced respiratory arrest was mitigated by the administration of a 5-HT2a agonist [50], suggesting that mortality can be reduced by stimulating the serotonergic system. In another genetic mouse model, LoF mutations in the 5-HT2c receptor were associated with epileptic seizures and spontaneous deaths resulting from seizures [47]. Therefore, normal functioning of both 5-HT2a and 5-HT2c receptors appears to be critical for preventing seizure-induced death.

Pharmacological augmentation of serotonergic signaling through the administration of 5-HT reuptake inhibitors (SRIs) has been shown to have preventative effects on seizure-induced respiratory arrest in different mice models, including DBA/1 [165,166,167,168], DBA/2 [48], and MES-induced seizures [50]. In humans, SRIs decreases the incidence of ictal central apnea in epileptic patients admitted to epilepsy monitoring unit [169] and reduces SUDEP incidence in DS patients [170]. Also, there is a potential for SRI to augment interictal HCVR in epileptic patients with a high risk of SUDEP [171] to reduce severity of ictal hypoxemia in medically refractory partial epilepsy [172]. These findings are supported by evidence that the loss of serotonergic signaling through the administration of a 5-HT antagonist, cyproheptadine, has been shown to do the opposite and increase the likelihood of seizure-induced respiratory arrest in a DBA/2 mice [48]. SRIs do not only improve the respiratory mechanisms but also have anticonvulsive properties. In mice, SRIs were shown to decrease susceptibility to seizures [173] and are currently under clinical trial to be used in human epileptic subjects as an anticonvulsant [174].

These studies indicate that the serotonergic system is compromised during fatal seizures and that the chemosensitive properties of raphe neurons may be impaired in epileptic conditions, potentially contributing to central apnea and respiratory arrest in SUDEP. However, evidence suggests that the chemosensitivity of raphe may be limited to only a small subset of neurons [175,176,177]. For example, in a study on anesthetized rats, the majority of tested 5-HT neurons (24 neurons) showed no electrical activity or responsiveness to severe hypercapnia [145]. Additionally, neurotoxic lesioning of medullary raphe neurons in piglets had no effect on the CO_2_ ventilatory response [178]. These point to inconsistencies in the evidence surrounding the chemosensitive properties of 5-HT neurons. Nevertheless, some studies support an additive interaction between raphe and RTN neurons in mediating central respiratory chemoreflexes. For instance, focal CO_2_ dialysis in the raphe obscurus has been shown to enhance the response to focal CO_2_ dialysis in the RTN, suggesting a facilitatory role of 5-HT neuron projections to the RTN [179,180]. Furthermore, there is evidence of a synergistic interaction between RTN and 5-HT raphe neurons; pharmacological lesioning of the RTN or the raphe alone reduces minute ventilation by 24% and 2.5%, respectively, while combined lesioning of both areas leads to a 50.7% reduction [181]. These findings suggest that a network of nuclei may be involved in the respiratory pathophysiology of SUDEP. For instance, the protective effect of SRI on seizure-induced respiratory arrest depends on serotonergic projections to the RTN, which are diminished in WARs with reduced HCVR [99]. While studies on specific neuron populations provide valuable insights, a more comprehensive understanding of the central chemoreceptor circuitry is crucial to better address respiratory symptoms in epilepsy.

### 6.3. Nucleus of the Solitary Tract

The caudal NTS is a critical neural structure that orchestrates respiratory and sympathetic functions by integrating signals from baroreceptors and chemoreceptors to generate homeostatic reflex responses [182,183,184,185]. While the NTS is well established as a mediator of peripheral chemoreceptor effects on respiratory control [186], its role in central chemoreception remains controversial. Some studies suggest that NTS neurons exhibit increased firing rates in response to physiological acidification [187,188], with NTS lesions or blockades leading to attenuated HCVR [188,189]. Conversely, other studies have not observed increased c-Fos expression in NTS neurons following CO_2_ exposure [190,191], and NTS lesions have sometimes been associated with heightened HCVR [192].

At the neuronal level, the NTS contains a heterogeneous population of neurons, including Phox2b+, glutamatergic, GABAergic, and TH+ neurons. Among these, Phox2b+, glutamatergic, and TH+ neurons are proposed to be chemosensitive, responding directly to CO_2_ increases [187,193,194]. Studies using epileptic animal models, such as kainic acid-injected rats, have observed a general decrease in neuronal populations within the NTS, a loss that becomes more pronounced with time and could weaken the NTS’s chemosensitive functions [195]. While the exact phenotype of these neurons remains undetermined, postmortem analyses in SUDEP patients have not revealed significant reductions in TH+ neuron populations [78].

Additionally, the responsiveness of NTS neurons (c-Fos immunohistochemistry) to CO_2_ is diminished in ARK rats with ablated HCVR [101], while other models, such as WARs, show no significant differences in Phox2b+ neuron populations or their CO_2_ responsiveness compared to the controls [99]. This discrepancy could be due to the different models used in these studies. Taking into account the essential function of the NTS in initiating integrative reflex responses, there is a need for thorough profiling of chemosensitive neurons and how these behave in pathological conditions like epilepsy, potentially predisposing individuals to SUDEP.

Similar to the RTN, the NTS expresses Kv1.1 proteins [153] and *Kcnq2/Kcnq3* mRNA in Phox2b+ neurons [61], suggesting that it may also serve as a substrate for genetic epilepsies involving *Kcna1* and *Kcnq2* mutations. Such mutations and the associated chemosensitive impairments may contribute to respiratory dysregulation in SUDEP. In *Kcna1* KO mice, extensive astrocytosis and microgliosis have been observed in the NTS [153], suggesting that seizure-related injuries might exacerbate respiratory dysfunctions. Detailed characterization of ion channels in the NTS could thus provide insights into its excitability and functional role during epileptic events.

Also, the seizure-induced activation of the NTS has been implicated in impaired arousal and gasping behavior, which could increase the risk of respiratory failure during seizures [196]. Notably, increased excitability of inhibitory GABAergic neurons in the NTS has been observed in epileptogenesis of pilocarpine mice [88], suggesting that alterations within NTS circuitry may disrupt the normal balance of excitatory and inhibitory signaling. Such dysregulation may contribute to respiratory instability and predispose individuals to SUDEP by compromising the NTS’s integrative and chemosensitive capabilities.

### 6.4. Locus Coeruleus

The LC is the primary cluster of noradrenergic neurons in the pons and plays a crucial role in promoting arousal. Studies on unanesthetized rats indicate that localized acidosis in the LC, induced by acetazolamide injection, increases ventilation [197,198], and the targeted deletion of LC neurons decreases the HCVR, particularly during wakefulness and non-rapid eye movement (NREM) sleep [197,199]. Experiments in anesthetized animals and brain slices reveal that LC neurons are moderately activated by hypercapnia [200,201], supporting the idea that the LC contributes to respiratory chemosensitivity. These studies collectively demonstrate the chemosensitive potential of LC neurons across various experimental models. Moreover, LC neurons receive synaptic input from other CCR populations as well [202,203,204,205,206]. Thus, the LC’s response to CO_2_ may be partially mediated by these chemosensory connections. However, it remains uncertain whether the selective activation of LC neurons directly stimulates respiration in vivo.

In epilepsy, LC neurons exhibit enhanced responsiveness to CO_2_, whereas responsiveness in other chemosensitive areas, such as the RTN, serotonin neurons, and the NTS, is diminished in the ARK and WAR rat models with attenuated HCVR [101]. Interestingly, in disease models like Parkinson’s disease, LC neurons become more responsive to hypercapnia when RTN neurons are impaired [207], suggesting that the LC may act as a compensatory mechanism when other chemosensitive areas lose CO_2_ sensitivity. Additionally, similar to the RTN and NTS, the LC expresses *Kcnq2/Kcnq3* mRNA in Phox2b+ neurons, suggesting it may serve as a substrate for genetic epilepsies associated with *Kcnq2* mutations [61]. These mutations, along with respiratory chemosensitivity changes in the LC, could contribute to the respiratory dysregulation observed in SUDEP.

The LC is also the main site for noradrenaline synthesis in the brain, and noradrenergic modulation of respiration is significant in epilepsy. Research involving a neurotoxin that selectively targets the noradrenergic transporter and destroys LC-originating terminals has demonstrated the importance of LC noradrenergic neurons in preventing seizure-induced respiratory arrest [208]. Similarly, in DBA mice exposed to audiogenic stimulation and pentylenetetrazol injections, post-seizure respiratory arrest was associated with reduced TH enzyme activity, resulting in decreased noradrenaline synthesis in the brainstem [209]. Other studies involving the chemical ablation of LC neurons have shown that the protective effects of SRIs on respiration during seizures depend on noradrenaline [210]. Hence, noradrenalin is an important target to reduce seizure-induced respiratory arrest in SUDEP.

During seizures, there is a consistent release of noradrenaline [211]. Knowing the role of the LC in the modulation of behavioral arousal [183,212], perhaps the LC is activated during seizures to induce arousal from apnea. Moreover, vagus nerve stimulation, an effective therapy for refractory epilepsy, is thought to reduce seizures by increasing noradrenaline synthesis in the LC [213]. Although the precise mechanisms by which LC activation exerts antiseizure effects are not fully understood, its roles in regulating behavioral arousal, chemosensitivity, and respiratory control [214] underscore the complexity of the LC’s involvement during epileptic events.

### 6.5. Hypothalamus

Some studies suggest that wake promoting orexinergic neurons in the hypothalamus are chemosensitive to elevated CO_2_ levels. These neurons are activated by acidification in vitro [215], and a small fraction exhibit increased c-Fos expression following hypercapnia in the perifornical region and the dorsomedial hypothalamus [206]. Furthermore, prepro-orexin KO mice, as well as healthy mice and rats treated with orexin receptor antagonists, exhibit decreased HCVR [216,217,218,219]. These findings highlight the role of orexinergic neurons in the hypothalamus as central mediators of CO_2_ responsiveness. Indeed, neurons in the LHA have been shown to sense extracellular pH changes via proton-sensing channels and regulate respiration by projecting to the NTS [220]. Orexinergic projections from the LHA to the VRC have also been identified [221], although it remains unclear whether these projections originate from chemosensitive neurons.

In epileptic conditions, hypothalamic seizures have been shown to disrupt brainstem mechanisms, leading to severe autonomic responses, including respiratory failure and acidosis [222]. This implies that seizure-induced hypothalamic dysregulation can disturb blood CO_2_ homeostasis, pointing to abnormalities in central chemoreception. Orexinergic signaling appears to increase in epileptic situations. In a pilocarpine-induced TLE rat model, there is selective activation of orexin neurons during seizures [223]. Similarly, high-risk *Kcna* KO mice exhibit an increased number of orexin neurons in the LHA [123]. Ex vivo extracellular recordings from the LHA of *Kcna* KO mice further demonstrate that orexin neurons are more responsive to reduced pH compared to controls [139]. These mice also display enhanced HCVR, as well as hypopnea/apnea and intermittent bradycardia, which are normalized with pretreatment using orexin receptor antagonists [123,139]. Consequently, orexin antagonists are being considered as therapeutic interventions for SUDEP-related cardiorespiratory symptoms [123]. However, because orexinergic neurons respond to hypercapnia [206], blocking their activity could worsen HCVR and increase SUDEP risk [216,218,219]. Also, while *Kcna* KO mice display increased HCVR, most of the other epileptic animal models are characterized by decreased HCVR (Table 2), which is thought to be a precursor to respiratory arrest in SUDEP. Thus, further investigation into orexinergic signaling in various animal models with a high risk of SUDEP is essential to clarify its role under different conditions in order to use it as a therapeutic target.

Additionally, it should be noted that orexinergic projections target various cardiorespiratory-related regions, including other CCRs (RTN, NTS, medullary raphe) and non-CCRs (VRC, periaqueductal gray, rostral VLM, hypoglossal nucleus, parabrachial/Kölliker–Fuse complex, and phrenic nuclei) [224]. Focal antagonism of the orexin receptor 1 in CCR sites, such as the RTN or the medullary raphe, reduces the CO_2_ response predominantly during wakefulness [218,219]. Collectively, these findings suggest the existence of a complex orexinergic circuitry that interacts with brainstem populations to regulate chemorespiratory functions. This suggests that its role extends beyond central chemoreception, and maintaining orexin homeostasis may be critical in managing epilepsy-induced autonomic disturbances.

**Table 4:** Mechanisms of disrupted central chemoreception in SUDEP.

CCR	Species	Model	Finding	References
RTN	Rat	Wild type	KCNQ antagonists increased and KCNQ agonist silenced CO_2_ responsiveness of RTN neurons in vitro	[154]
		WAR	Decreased number of RTN neurons and decreased activation of RTN neurons in response to hypercapnia	[99]
		ARK	Decreased activation of RTN neurons in response to hypercapnia	[101]
	Mouse	Dravet Syndrome (*Scn1a* missense mutation in inhibitory neurons)	Altered electrical activity of the RTN in response to CO_2_/H+ in DS mice in brainstem slices	[59]
		Kainic acid (intrahippocampal)	Abolished firing profile of excited adapted neurons in response to CO_2_ in the RTN but not in the RVLM (Ca^2+^ imaging in freely moving)	[60]
		Wild type	Kv1.1 protein encoded by epilepsy related gene *Kcna1* is expressed the RTN	[153]
		*Kcna1* KO	*Kcna1* KO is associated with significant astrocytosis and microgliosis in the RTN	[153]
		Phox2b^Cre/+^::Ai14 mice (TdTomato reporter expressed in Phox2b neurons)	The RTN expresses *Kcnq2* but not *Kcnq3* transcripts	[61]
		*Kcnq2* GOF variant R201C in Phox2b+ neurons	Decreased HCVR during the light/inactive state with mutation of *Kcnq2* in the RTN	[61]
Raphe	Human	SUDEP cases	Decreased TPH2 and SERT in medullary raphe and VLM	[80]
		SUDEP cases	Atrophy in raphe	[159,160,161]
		Epileptic patients admitted to epilepsy monitoring unit	SRIs decreases the incidence of ictal central apnea	[169]
		Dravet syndrome	SRIs reduces SUDEP incidence	[170]
		Epileptic patients with high risk of SUDEP	SRIs augment interictal HCVR	[171]
		Medically refractory partial epilepsy	SRIs reduce severity of ictal hypoxemia	[172]
	Rat	Intrahippocampal electrical stimulation	Suppression of 5-HT neurons in the dorsal and medullary raphe during ictal and postictal periods in concert with decreased respiratory rate, tidal volume, and minute ventilation	[164]
		WAR	Decreased number of 5-HT neurons and decreased activation of 5-HT neurons in response to hypercapnia	[99]
		ARK	Decreased activation of 5-HT neurons in response to hypercapnia	[101]
		Pilocarpine	A sharper decrease in oxygen consumption with decreased 5-HT levels in the NTS and VLM Increased transcript levels for TPH2 and SERT in the brainstem	[136]
	Mouse	5-HT2C mutant mice	Spontaneous seizures with deaths	[47]
		Lmx1bf/f/p	Impaired HCVR	[163]
		Lmx1bf/f/p	Impaired arousal response to hypercapnia during sleep	[56,57]
		Lmx1bf/f/p	Decreased seizure threshold in response to maximal electroshock Increased rate of seizure-induced mortality due to respiratory failure mitigated by the administration of a 5-HT2a agonist	[50]
		DBA/1	Increased activity of raphe nuclei associated with audiogenic seizure-induced respiratory arrest	[96]
		DBA/1	SRI decreases seizure-induced respiratory arrest	[165,166,167,168]
		DBA/2	SRI decreases seizure-induced respiratory arrest	[48]
		DBA/2	5-HT antagonist, cyproheptadine, increase the likelihood of seizure-induced respiratory arrest	[48]
NTS	Rat	Kainic acid	Decreased neuronal populations within the NTS more pronounced with time	[195]
		Kainic acid	Seizure-induced activation of the NTS associated with impaired arousal and gasping behavior	[196]
		ARK	Decreased activation of NTS neurons in response to hypercapnia	[101]
	Mouse	Wild type	Kv1.1 protein encoded by epilepsy related gene *Kcna1* is expressed the NTS	[153]
		*Kcna1* KO	*Kcna1* KO is associated with significant astrocytosis and microgliosis in the NTS	
		Phox2Cre/+::Ai14 mice (TdTomato reporter expressed in Phox2b neurons)	The NTS expresses *Kcnq2* and *Kcnq3* transcripts	[61]
		*Kcnq2* GOF variant R201C in Phox2b+ neurons	Decreased HCVR during the light/inactive state with mutation of *Kcnq2* in the NTS	[61]
LC	Rat	ARK	Increased activation of LC neurons in response to hypercapnia	[101]
	Mouse	Phox2Cre/+::Ai14 mice (TdTomato reporter expressed in Phox2b neurons)	The LC expresses *Kcnq2* and *Kcnq3* transcripts	[61]
		*Kcnq2* GOF variant R201C in Phox2b+ neurons	Decreased HCVR during the light/inactive state with mutation of *Kcnq2* in the LC	[61]
		DBA/1	Neurotoxin ablation of LC-originating terminals prevents seizure-induced respiratory arrest	[208]
		DBA/1 (audiogenic stimulation and pentylenetetrazol)	Respiratory arrest associated with reduced TH enzyme activity	[209]
		MES in C57Bl6, Lmx1bf/f/p, NE deficient	The protective effects of SRIs on respiration during seizures depend on noradrenaline	[210]
Hypothalamus	Rat	Penicillin-G at hypothalamic and mesencephalic level	Hypothalamic seizures are characterized with acidosis	[222]
		Pilocarpine	Selective activation of orexin neurons during seizures	[223]
	Mouse	*Kcna* KO	Overstimulation of orexin neurons in response to reduced pH and increase HCVR HCVR normalized with orexin receptor antagonist	[139]
		*Kcna* KO	Orexin receptor antagonists decrease hypopnea/apnea and intermittent bradycardia	[123]

## 7. Neuropeptides Involved in Respiratory Dysregulation in Epilepsy

Research on the respiratory network has provided significant insights into the neuromodulators that regulate its function [225]. Neuropeptides are important to consider, as they are suggested to play an important role in modulating seizures and epilepsy [226]. Unlike neurotransmitters, which operate on a millisecond timescale, neuropeptides have longer half-lives; enabling them to modulate neuronal and network activity over extended periods [227,228]. This property contributes to setting the seizure threshold and endows them with anticonvulsive properties. Beyond their ability to reduce the likelihood of seizures, neuropeptides are essential for breathing regulation, as they are expressed in CCRs (Figure 3). Consequently, their malfunction may lead to respiratory depression and impairment of central chemosensitive networks in SUDEP.

### 7.1. Galanin

For instance, postmortem studies of SUDEP patients have revealed a reduction in the number of galanin terminals in the VLM [80]. Galanin, expressed in subpopulations of chemosensitive neurons in the RTN, NTS, LC, and hypothalamus, is known to regulate breathing by sustaining chemoresponsiveness and altering gene expression in response to hypercapnia [229]. Experimental evidence suggests that galanin administration protects against seizure-induced respiratory arrest in mice [230]. In contrast, galanin receptor antagonists in the LC have been shown to improve impulsive-like behaviors in epileptic rat models [231]. Galanin analogs are proposed as potential anti-seizure medication [232,233,234,235]. Galnon or Galmic are synthetic non-peptide galanin receptor agonists capable of crossing the blood–brain barrier and reducing pentylenetetrazol-induced seizures in rodents [236,237,238]. However, these analogs have yet to be tested in human clinical trials [238]. Hence, galanin can be a candidate in contributing to seizure-induced respiratory dysfunction.

### 7.2. PACAP

Similarly, PACAP is a neuropeptide found in the brainstem centers that are critical for central cardiorespiratory regulation [239]. In kainic acid-induced rats, intrathecal PACAP antagonists exacerbate seizure-induced sympathoexcitation, resulting in increased blood pressure and heart rate [240]. This suggests that PACAP plays a protective role against the adverse cardiovascular effects of seizures. PACAP mRNA is also expressed in several central chemoreceptive regions, including the RTN, NTS, LC, and LHA rate [241,242]. Notably, its expression is upregulated in the hypothalamus following kainic acid-induced seizures in rats [243]. PACAP knockout mice exhibit respiratory control defects, with PAC1 receptor deficiencies impairing cardiorespiratory responses to hypercapnia and hypoxia [244]. Neonatal PACAP-deficient mice are more susceptible to sudden death and show reduced respiratory responses to hypoxia and hypercapnia [245]. Furthermore, restoring PACAP expression in RTN neurons of PACAP-deficient mice improves breathing responses to CO_2_ and reduces apneas [150]. Collectively, these findings indicate that PACAP may protect against SUDEP-related central chemoreception impairment.

### 7.3. Orexin

Another neuropeptide of interest to mention in this context is orexin. As mentioned in the previous section, orexinergic neurons in the hypothalamus are sensitive to CO_2_, and their signaling increases in epilepsy [123,223,246]. Orexin also plays a vital role in sleep-state-dependent breathing regulation and shows a pronounced diurnal variation. Studies in rats demonstrate that orexin levels are highest at the end of the wake-active period, with even stronger fluctuations observed in older animals [247,248]. Orexin-deficient mice and those treated with orexin receptor antagonists exhibit attenuated HCVR during wakefulness but not during sleep, suggesting vigilance-state specificity [216,217,218,219]. The intracerebroventricular administration of orexin agonists partially restores these defects [249]. Orexin-deficient mice show frequent sleep apneas [217], and decreased orexin levels have been identified in patients with obstructive sleep apnea [250]. Orexin receptor antagonists are shown to improve sleep [251,252]. These studies highlight the potential involvement of orexin in the elevated risk of impaired chemosensory function during specific vigilance states, contributing to the increased risk of SUDEP during sleep.

### 7.4. Somatostatin

Somatostatin is another neuropeptide that plays a crucial role in central chemoreception, acting as an inhibitory modulator of respiration. Single-cell RNA sequencing studies have identified somatostatin-expressing interneurons within the parafacial region, where the RTN is located [253]. These somatostatin-positive interneurons are CO_2_ sensitive but paradoxically appear to be inhibited by CO_2_ [253]. In vitro experiments suggest that RTN neurons receive inhibitory input from these nearby parafacial somatostatinergic neurons. Notably, in vivo studies have demonstrated that the selective chemogenic inhibition of somatostatin-containing parafacial neurons enhances baseline respiration [253]. This indicates that CO_2_ not only directly stimulates RTN neurons but may also facilitate their disinhibition by suppressing somatostatin signaling. Further supporting this role, studies in rats have shown that somatostatin administration in the VLM blunts the ventilatory response to both hypoxia and hypercapnia [253]. In contrast, human studies have found that intravenous somatostatin infusion significantly reduces the ventilatory response to hypoxia while leaving the response to hypercapnia unchanged [254,255]. Importantly, a postmortem analysis of SUDEP brain samples has revealed a significant reduction in somatostatin labeling in the VLM compared to the controls [80]. Collectively, these findings suggest that alterations in somatostatin signaling may contribute to impaired chemosensory function within the VLM, potentially playing a role in SUDEP pathophysiology.

### 7.5. Bombesin-Like Peptides

The RTN contains several other neuropeptides, including bombesin-like peptides called neuromedin B (NMB) and gastrin-releasing peptide (GRP) [242,256]. RTN neurons exhibit increased NMB and GRP mRNA expression in response to short-term CO_2_ exposure [229]. NMB neurons also show elevated Fos mRNA levels following acute hypercapnia [242]. These findings highlight the role of these neuropeptides in central chemoreception. Beyond chemoreception, NMB and GRP neurons in the RTN are involved in the sighing mechanism [256]. Sighs—long, deep breaths—occur in response to emotional and physiological stressors, such as hypoxia and hypercapnia [257], enhancing gas exchange and maintaining lung integrity by reinflating collapsed alveoli [256]. Following clonic seizures, patients commonly release a deep sigh before resuming normal breathing and arousal, which may serve as a protective mechanism against SUDEP-associated respiratory arrest. Although NMB and GRP have demonstrated anticonvulsant properties [258], their precise role in central chemoreception and sighing, particularly in the context of SUDEP, remains to be fully elucidated.

In summary, the neuropeptides galanin, PACAP, orexin, somatostatin, and bombesin-like peptides play critical roles in central chemoreception, respiratory regulation, and seizure modulation. These neuropeptides contribute to maintaining respiratory homeostasis by influencing central chemosensory networks and cardiorespiratory control centers. Their dysregulation may result in impaired respiratory responses, chemosensory dysfunction, and autonomic instability, which are associated with conditions like SUDEP. Understanding the complex roles of these neuropeptides provides valuable insights into potential therapeutic targets for mitigating respiratory and seizure-related risks.

## 8. Research Perspectives and Diagnostic and Therapeutic Implications

This review underscores the pivotal role of central chemoreception in the pathophysiology of SUDEP (Figure 3), highlighting potential future diagnostic and therapeutic approaches, such as the identification of novel candidate biomarkers for SUDEP risk and to the development of new preventive treatments. The targeting of specific CCRs, including the RTN, raphe nuclei, NTS, LC, and hypothalamus, pharmacologically holds promise for stabilizing respiratory function in epilepsy patients. There exist initial pilot studies that explored the efficacy of a serotonin reuptake inhibitor, fluoxetine, to augment HCVR in epilepsy patients [171]. Other therapeutic agents targeting CCR regulatory candidates, including neuropeptides—particularly galanin, PACAP, orexin, somatostatin, and bombesin-like peptides—could be tested to enhance ventilatory responses to hypercapnia and potentially reduce SUDEP risk.

The therapeutic development of these agents should consider advanced techniques such as gene therapy and CRIPSR/dCas9 technology. Currently, these innovative approaches are emerging in the field of epilepsy. Among genetic epilepsies (e.g., *Kcna1*, *Kcnq*, *Scn1a* genes), the *Scn1a* mutation, responsible for DS, has been the primary target. An adeno-associated virus (AAV) capsid called ETX101 is currently being tested [259] (ClinicalTrials.gov Identifier: NCT05419492 in the USA, NCT06283212 in the UK and NCT06112275 in Australia). ETX101 is administered via intracerebroventricular infusion and delivers a transgene encoding an engineered SCN1A-specific transcription factor within GABAergic interneurons to upregulate the endogenous SCN1A gene expression and thereby increasing NaV1.1 protein levels [260,261]. Additional engineered vectors in clinical trials include a lentiviral vector that delivers a potassium channel (EKC) to excitatory neurons [262] (ClinicalTrials.gov Identifier: NCT04601974) and another AAV vector, AMT-260, which encodes microRNA that block the mRNA encoding glutamate receptor, GluK2, and thereby downregulates its expression [261,263] (ClinicalTrials.gov Identifier: NCT06063850). Both EKC and AMT-260 are administered through intracerebral infusion into the target area. The CRIPSR/dCas9 (Clustered Regularly Interspaced Short Palindromic Repeats-CRISPR associated protein) approach is a powerful tool for genome editing [264] but relatively new in the field of epilepsy. Transcriptional activators fused to a dCas9 have been shown to alter *Scn1a* gene expression [265,266]; however, no clinical trials exist yet. These advancements could inspire new treatments targeting other SUDEP-related pathways.

Beyond therapeutic interventions, impaired CO_2_ handling could serve as a biomarker to identify individuals at elevated risk for SUDEP, offering a valuable predictive tool for clinicians. HCVR can be performed in epileptic patients in the EMU using a modified hyperoxic rebreathing technique [58,81]. This technique has shown promise as a rapid and safe diagnostic tool for epilepsy patients [81]. Early studies observed significant interindividual differences in HCVR or central respiratory CO_2_ chemosensitivity [58,81]. Notably postictal HCVR measurements have revealed prolonged impairment in CO_2_ chemoreception after seizures [58]. Moreover, longitudinal HCVR assessments are also important to determine whether CO_2_ chemosensitivity deteriorates over time, aiding in risk prediction for SUDEP [267]. Hence, HCVR measurements (longitudinal, interictal and postictal) are promising in predicting the risk of breathing abnormalities like hypoventilation and hence identify patients who are at increased risk for SUDEP. However, to maximize its clinical utility, standardized HCVR testing protocols must be established and integrated into routine epilepsy care.

Despite these favorable findings, the clinical integration of HCVR remains limited. Although the recent SUDEP-CARE score [22] incorporates respiratory symptoms during or after seizures as a critical risk factor, HCVR has yet not been directly included in SUDEP risk assessment protocols. Standardizing HCVR testing and integrating it into routine epilepsy care will require large-scale, multicenter, prospective, and longitudinal studies to validate its efficacy as a biomarker.

Once validated in clinical settings, HCVR could serve as a proxy biomarker or an outcome measure to assess the effectiveness of SUDEP prevention strategies. This approach could address the challenge of the long-term monitoring of a large number of epilepsy patients who are at risk of SUDEP, providing quicker insights into the impact of proposed interventions. However, it is essential to recognize that SUDEP is influenced by multifactorial mechanisms. Beyond a dysfunction at the central respiratory chemoreception, factors such as genetic predisposition, cardiac abnormalities, impaired arousal mechanisms, non-compliance to medication, and environmental triggers also contribute to SUDEP risk. Thus, a comprehensive framework encompassing multiple outcome measures—including HCVR, seizure frequency and severity, cardiac parameters, and autonomic responses—is essential for evaluating new therapeutic agents.

## 9. Conclusions

Respiratory dysfunction, particularly hypoventilation, is a significant contributing factor to SUDEP. Emerging evidence indicates that CO_2_ imbalance during and after seizures reflects impairments in central chemoreception mechanisms. HCVR as a method to assess CO_2_ sensitivity holds potential for identifying patients at heightened risk for SUDEP. Advancing our understanding of central respiratory chemoreception in epilepsy not only offers insights into SUDEP pathophysiology but also paves the way for innovative pharmacological interventions. These efforts could significantly reduce the burden of SUDEP and improve outcomes for epilepsy patients.

## Figures and Tables

**Table 3:** Suggested involvement of increased central respiratory CO_2_ chemosensitivity in SUDEP.

Species	Model	Condition	Epileptogenesis	Evidence	Reference
Rats	Pilocarpine Wistar (intraperitoneal)	Chronic unanesthetized	4–8 weeks after first spontaneous seizure	Decreased in the latency to awaken from obstructive sleep apneas during REM sleep	[135]
	Pilocarpine Sprague-Dawley (intraperitoneal)	Chronic unanesthetized	12 weeks post-SE	Low steady respiratory pattern (breathing rhythm) but enhanced ventilatory response to hypoxia and hypercapnia in	[136]
Mouse	*Kcna* KO	Chronic unanesthetized	P49	Increased HCVR	[139]
